# Characteristics of the use of dexmedetomidine in critically ill children: a Brazilian study

**DOI:** 10.1186/cc14568

**Published:** 2015-03-16

**Authors:** PL Lago, C Andreolio, J Piva, E Baldasso

**Affiliations:** 1 Hospital de Clinicas de Porto Alegre, Brazil

## Introduction

To describe the main indications, doses, infusion length and side effects of dexmedetomidine (DEX) administered to children and adolescents admitted to the pediatric ICU (PICU).

## Methods

A retrospective observational study including children (<18 years) admitted to a Brazilian PICU who received DEX between November 2011 and June 2014. Demographic data, indications, initial dose, maximum dose and time of infusion of DEX, side effects and impact on heart rate (HR) and mean arterial pressure (MAP) 6 and 24 hours after the start of infusion.

## Results

A total of 77 children with a median age of 15 (4 to 84) months, weight of 10 (5.7 to 20) kg and length of ICU stay of 8 (5 to 14) days received DEX, with a mortality rate of 9%. Indications were: weaning from mechanical ventilation (32.5%), neurosurgical postoperative (NCI) and upper airway surgery (VAS) (24.7%), non-invasive ventilation (13%), refractory tachycardia (6.5%) and other indications (23.3%). There was no difference between the initial and maximum doses and infusion length. There was a significant decrease in MAP and HR after 6 hours infusion of DEX in the total group; however, no significant difference occurred between groups when analyzing MAP and HR 24 hours after the start of infusion (*P *= 0.798 and 0.379, one-way ANOVA, respectively). In six patients (8%) DEX was suspended for possible side effects.

## Conclusion

Increased DEX indications have been observed in the pediatric population. In this study DEX was demonstrated to be a safe and tolerable drug with few side effects, especially related to the cardiovascular system.
